# Identification of Monocyte-Associated Genes Related to the Instability of Atherosclerosis Plaque

**DOI:** 10.1155/2022/3972272

**Published:** 2022-09-21

**Authors:** Wentao Qin, Fu Gan, Riguan Liang, Jing Li, Xiaomei Lai, Yongfa Dai, Jie Liu

**Affiliations:** ^1^Department of Bone and Joint, The First Affiliated Hospital of Guangxi Medical University, Nanning, Guangxi 530021, China; ^2^Department of Urology Surgery, The Affiliated Hospital of Youjiang Medical University for Nationalities, Baise, China; ^3^Department of Joint Surgery and Sports Medicine, Baise People's Hospital, Baise 533000, China; ^4^Department of Nuclear Medicine, The First Affiliated Hospital of Guangxi Medical University, Nanning, Guangxi 530021, China; ^5^Department of Cardiovascular Medicine, The First Affiliated Hospital of Guangxi Medical University, Nanning, Guangxi 530021, China; ^6^Department of Cardiovascular Medicine, The First People's Hospital of Nanning, Nanning, Guangxi 530016, China; ^7^Department of Cardiovascular Medicine, The Fifth Affiliated Hospital of Guangxi Medical University, Nanning, Guangxi 530016, China

## Abstract

**Background:**

Atherosclerotic plaque instability is a common cause of stroke and ischemic infarction, and identification of monocyte-associated genes has become a prominent feature in cardiovascular research as a contributing/predictive marker.

**Methods:**

Whole genome sequencing data were downloaded from GSE159677, GSE41571, GSE120521, and GSE118481. Single-cell sequencing data analysis was conducted to cluster molecular subtypes of atherosclerotic plaques and identify specific genes. Differentially expressed genes (DEGs) between normal subjects and patients with unstable atheromatous plaques were screened. Weighted gene coexpression network analysis (WGCNA) was performed to find key module genes. In addition, GO and KEGG enrichment analyses explored potential biological signaling pathways to generate protein interaction (PPI) networks. GSEA and GSVA demonstrated activations in plaque instability subtypes.

**Results:**

239 monocyte-associated genes were identified based on bulk and single-cell RNA-sequencing, followed by the recognition of 1221 atherosclerotic plaque-associated DEGs from the pooled matrix. GO and KEGG analyses suggested that DEGs might be related to inflammation response and the PI3K-Akt signaling pathway. Eight no-grey modules were obtained through WGCNA analysis, and the turquoise module has the highest correlation with unstable plaque (*R*^2^ = 0.40), which contained 1323 module genes. After fetching the intersecting genes, CXCL3, FPR1, GK, and LST1 were obtained that were significantly associated with plaque instability, which had an intense specific interaction. Monocyte-associated genes associated with atherosclerotic plaque instability have certain diagnostic significance and are generally overexpressed in this patient population. In addition, 11 overlapping coexpressed genes (CEG) might also activated multiple pathways regulating inflammatory responses, platelet activation, and hypoxia-inducible factors. GSVA showed that the corresponding pathways were significantly activated in high expression samples.

**Conclusions:**

Overexpression of CXCL3, GK, FPR1, and LST1 was advanced recognition and intervention factors for unstable plaques, which might become targets for atherosclerosis rupture prevention. We also analyzed the potential mechanisms of CEG from inflammatory and oxidative stress pathways.

## 1. Introduction

Atherosclerosis (AS) is a common cardiovascular disease with a high mortality and morbidity rate due to chronic inflammatory changes caused by the accumulation of cholesterol on the walls of blood vessels over time. Atherosclerosis mainly occurs in medium and large arteries, characterized by the formation of multifocal atherosclerotic plaques on the inner wall of arteries [1–4]. Atherosclerotic plaques are mainly composed of lipids, necrotic cells, and some inflammatory factors, which are divided into stable and unstable plaques according to the stability of the plaques. The rupture of unstable atherosclerotic plaques often leads to serious consequences, including ischemic stroke and acute myocardial infarction. Some studies have found that more than 70% of fatal coronary thrombosis is caused by the rupture of unstable plaques [5, 6]. In-depth elucidation of the molecular mechanism of unstable plaque formation to achieve early recognition and intervention is of great significance to prevent severe clinical complications. However, the specific molecular mechanisms involved in the instability of atherosclerotic plaques are very complicated and have not been fully reported. It is imperative to conduct in-depth research on it.

Currently, only a few studies have reported the possible factors involved in the formation and rupture of unstable plaques. For example, Bao et al.'s study suggested that CD5L, S100A12, and CKB were involved in regulating the stability of atherosclerotic plaques [7]. Other studies reported that visfatin could act as an inflammation mediator and ultimately aggravated the instability of atherosclerotic plaques [8, 9]. Liu et al. reported the downregulation of IGFBP6 expression in unstable carotid atherosclerotic plaques and indicated that it might be an available prediction marker for vulnerable plaques [10]. Besides, a previous study has confirmed that EFEMP1, BGN, and RILP may have potential diagnostic value for atherosclerotic plaque rupture [11]. Another study suggested that the stability of atherosclerotic plaques may be related to oxidative stress of cells, and the oxidative stress caused by excessive reactive oxygen species in cells plays an important role in atherosclerosis. In carotid plaques, SIRT6 can promote the occurrence of oxidative stress through various ways, and it is obviously overexpressed in unstable carotid plaques, suggesting that the process of oxidative stress is a dangerous factor in the stability of plaques. Summarily, many studies have identified various factors that may contribute to plaque instability. However, it is worth noting that the above research and analysis on plaque instability are mostly limited to the transcriptome level. Existing studies have confirmed the difference in cell composition between ruptured and stable plaques [12–14]. Also, the underlying mechanism of macrophages and foam cells involved in atherosclerotic plaque instability and rupture has been partially characterized [15, 16]. Numerous studies have reported that monocytes play an essential role in atherosclerotic, but monocyte-associated genes' effect on plaque instability is still not very clear [17–21]. Therefore, it is essential to explore the clinical significance and potential mechanisms of monocyte-associated genes in atherosclerotic plaque instability through the combined single-cell sequencing and transcriptomics data analysis.

In this study, we combined single-cell sequencing data analysis, differential expression analysis, and WGCNA analysis to obtain significant monocyte-associated candidate genes related to atherosclerotic plaque instability. Furthermore, we detected the expression status of the obtained candidate genes in monocytes and unstable plaques and explored its diagnostic significance. Finally, GSEA, GSVA, coexpression analysis, etc., were used to explore the underlying mechanism preliminarily.

## 2. Materials and Methods

### 2.1. Data Acquisition and Preprocessing

The datasets (single-cell sequencing data: GSE159677 with 6 qualified samples; transcriptome data: GSE41571, GSE120521, and GSE118481 with 11, 8, and 16 qualified samples, respectively) included in this study were all obtained from GEO databases (https://www.ncbi.nlm.nih.gov/geo/). The transcriptome data were proceeded by log2 conversion and normalization, then used for subsequent analysis. Due to the small sample size, the SVA software package is used to integrate GSE41571 and GSE120521 datasets into a merged dataset containing ten stable plaque samples and nine unstable plaque samples, and batch correction between the two datasets is also solved.

### 2.2. Single-Cell Sequencing Data Analysis

To obtain monocyte-associated genes in atherosclerotic plaque, the GSE159677 dataset, which contained 6 matched samples, was used for single-cell sequencing data analysis through the Seurat and SingleR packages. We integrated the data of 6 samples and performed data normalization. Hereafter, data quality control was carried out. Briefly, cells with less than 50 genes and more than 5 percent of mitochondrial genes will be removed. The number of highly variable genes was set at 1500. Principal component analysis (PCA) was performed on the top 1500 variable genes to visualize transcriptional variability. T-SNE method was used for further dimensional reduction of the principal components (*p* value < 0.05), and then, cells were clustered. Monocyte-related genes were extracted using the FindAllMarkers function.

### 2.3. Weighted Coexpression Network Analysis (WGCNA)

WGCNA analysis was performed on the GSE118481 dataset to find gene module genes significantly associated with plaque instability. An appropriate soft threshold was reasonably selected as the degree of scale independence reached 0.9. Besides, the min module size is set to 30, and modules with a similarity over 0.8 will be merged. Genes in modules that most significantly correlated with unstable plaque were considered crucial genes associated with plaque instability.

### 2.4. Identification of Significant Monocyte-Associated Candidate Genes Related to Plaque Instability

We performed differential expression analysis on the merged dataset using the limma package. |logFC| > 1 and adj.*p*-value < 0.05 were used as the threshold to identify differentially expressed genes (DEGs). LogFC < −1 and logFC > 1 represented genes downregulated and upregulated in unstable plaques, respectively. Overlapping genes identified by single-cell sequencing data analysis, differential expression analysis, and WGCNA analysis were considered to be significant monocyte-associated candidate genes related to plaque instability. These overlapping candidate genes will be used for further analysis.

### 2.5. Determination of Expression and Diagnostic Value of Candidate Genes

To determine the candidate genes' ability to distinguish the stable from unstable plaques, we performed receiver operating characteristic (ROC) curve analysis in the R software (version 3.6.3) using the pROC package (for analysis) and the ggplot2 package (for visualization). Subsequently, we measured the area under the ROC curve (AUC) to evaluate the candidate genes' diagnosing capability. Moreover, we also extracted the expression profile data of candidate genes and then drew the violin plot to determine their expression patterns.

### 2.6. Functional Enrichment Analysis of DEGs

The DAVID database (https://david.ncifcrf.gov/) was widely used in bioinformatics analysis [22]. In our study, we used the DAVID database to carry out functional enrichment analysis to gain an in-depth understanding of the potential mechanisms of DEGs in plaque instability. Briefly, we carried out two functional enrichment analyses, including Gene Ontology (GO) analysis and the Kyoto Encyclopedia of Genes and Genomes (KEGG) pathway analysis. The analysis results were shown in the form of bubble charts.

### 2.7. Gene Set Enrichment Analysis (GSEA)

It was critical to explore the as-yet-undiscovered pathways associated with plaque instability. Therefore, based on the merged dataset, we performed GSEA and result visualization using the R packages clusterProfile and enrichplot, respectively. For this analysis, we used “h.all.v7.4.symbols.gmt” as the reference gene set.

### 2.8. Identification and Functional Analysis of Overlapping Coexpressed Genes (CEGs) of Candidate Genes

Using the merged dataset, we performed coexpression analysis on candidate genes, and genes with a Pearson correlation coefficient *r* greater or equal to 0.70 were considered as CEGs. GeneMANIA (http://www.genemania.org) was an online database that provided functional analysis services [23]. In our study, GeneMANIA was implemented to explore the molecular function of overlapping CEGs. In addition, we analyzed the related genes in GeneMANIA database in STRING database (https://cn.string-db.org/) at protein level.

### 2.9. Functional Similarity Analysis of Candidate Genes and Correlation Analysis of PI3K-ATK Pathway-Related Genes

We used GoSemSim package to analyze the interaction between protein of the candidate genes and scored their similarities. The scores were used to judge the functional effect. Finally, we selected the gene with the highest score and made a correlation analysis with PI3KR5 and other candidate genes.

### 2.10. Gene Set Variation Analysis (GSVA) and Identification of Correlations between the Candidate Genes

GSVA was performed using the R package “GSVA” to identify signaling pathways associated with candidate genes. Specifically, we performed the above analysis by dividing the samples into high and low expression groups according to the median expression values of candidate genes. Besides, “h.all.v7.4.symbols.gmt” was the reference gene set of GSVA. Meanwhile, we calculated the Pearson correlation coefficient between the candidate genes based on the merged dataset to explore their relationship.

### 2.11. Statistical Analysis

Statistical analysis involved in this study was completed on the R (version 3.6.3) platform. Comparison of candidate gene expression in stable and unstable plaques was performed using independent sample *t*-tests. Unless otherwise stated, *p* values less than 0.05 were considered statistically significant.

## 3. Results

### 3.1. Extraction of Monocyte-Related Genes

**Figure 1:**
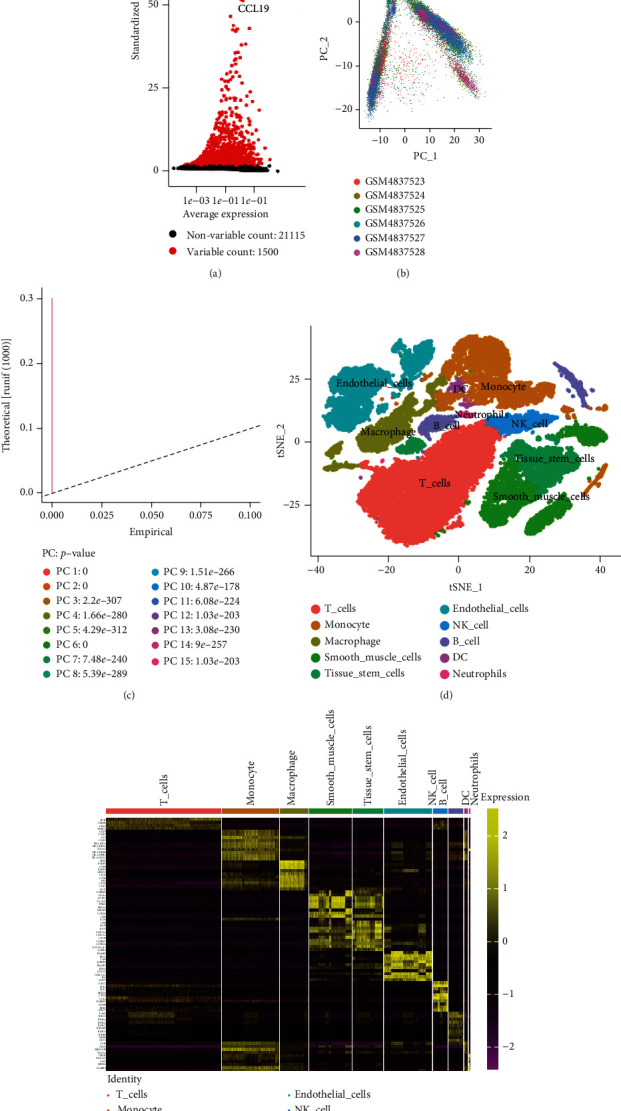
Extraction of monocyte-related genes by single-cell sequencing data analysis. (a) Gene filtering. (b, c) Principal component analysis (PCA) plot colored by various samples. (d) T-SNE plot colored by cell types. (e) Identification of various cell cluster-associated specific genes.

To obtain monocyte-related genes in atherosclerotic plaques, we first integrated and quality-controlled the single-cell sequencing data from the six samples included in GSE159677. The top 1500 variable genes are shown (Figure 1(a)). Further, the normalization of single-cell sequencing data was carried out, and 15 principal components (*p* value < 0.05) were used for subsequent analysis (Figures 1(b) and 1(c)). This study identified 10 cell clusters using the Seurat package, and cluster 2 was classified as monocytes (Figure 1(d)). We subsequently extracted cell cluster-associated specific genes and presented the top 10 genes for each cell cluster in a heatmap (Figure 1(e)). The extracted 239 monocyte-related genes will be used in subsequent studies.

### 3.2. Extraction of Genes Associated with Plaque Instability Phenotype

**Figure 2:**
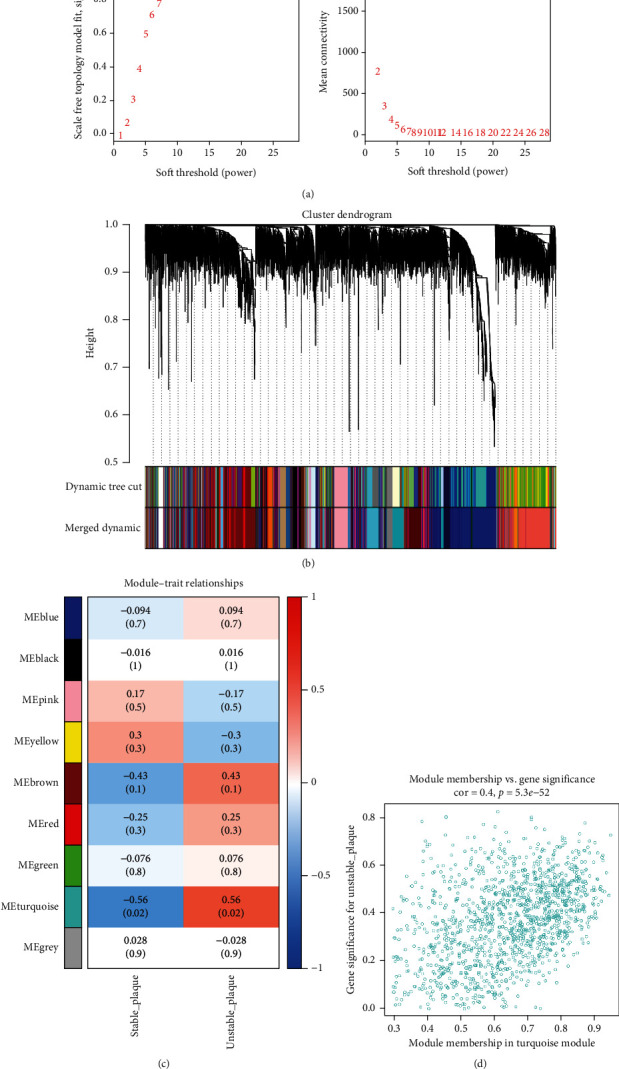
Identification of the crucial genes related to atherosclerotic plaque instability phenotype through weighted coexpression network analysis (WGCNA). (a) Scale-free fit parameters of different soft-thresholding powers and according to average connectivity of different soft-thresholding powers. (b) Cluster dendrogram obtained in this study. (c) The heatmap showed the correlation of modules and phenotypes and annotated the correlation coefficient and *p* value. (d) Gene significances of the selected turquoise module.

We utilized WGCNA analysis to identify modules significantly associated with the phenotype of unstable atherosclerotic plaques. Module genes were considered to be significantly associated with plaque instability. The parameters were set as follows: (1) MergeHeightCut = 0.25, (2) scale-free *R*^2^ = 0.9, (3) minimum size of module genes = 30, and (4) soft-thresholding power = 11. Ultimately, we obtained eight nongrey modules, among which the turquoise module containing 1323 genes had the strongest positive correlation with the unstable plaque phenotype (*R*^2^ = 0.40, *p*‐value = 5.3*e* − 52) (Figures 2(a)–2(d)). Genes included in this module will be selected for conducting further analysis.

### 3.3. Identification of 4 Significant Monocyte-Associated Candidate Genes Related to Plaque Instability

**Figure 3:**
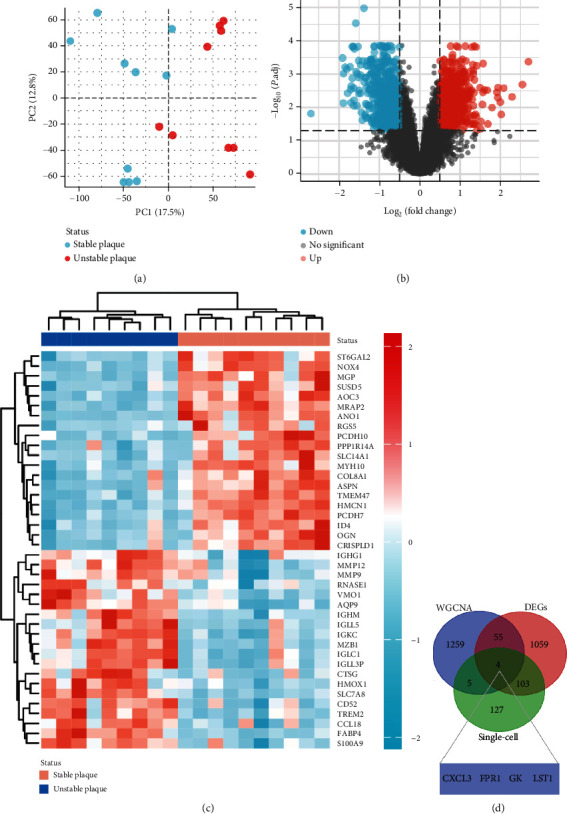
Differential expression analysis and identification of significant monocyte-associated candidate genes related to plaque instability. (a) Two-dimensional PCA cluster plot of the merged dataset; blue represented the stable sample, and red represented the unstable plaque sample. (b) Volcano plot of differentially expressed genes (DEGs), red represents upregulated genes, blue represents downregulated genes, and grey represents no significantly differentially expressed genes. (c) A heatmap of the most 20 upregulated and downregulated genes. (d) Venn diagram showed the obtained four overlapping significant monocyte-associated candidate genes (CXCL3, FPR1, GK, and LST1) related to plaque instability.

PCA analysis revealed significant differences in gene expression between stable and unstable plaques (Figure 3(a)). In our current study, a total of 1221 DEGs were identified from the merged dataset, which may play an important role in the development of unstable plaques (Figure 3(b)). The heatmap showed the top 20 upregulated and downregulated genes (Figure 3(c)). Only overlapping genes identified in single-cell sequencing data analysis, WGCNA analysis, and differential expression analysis will be considered significant monocyte-associated candidate genes related to plaque instability. We intersected the genes identified by the three approaches to obtain 4 overlapping genes, including CXCL3, FPR1, GK, and LST1, which will be used as candidate genes for follow-up research (Figure 3(d)).

### 3.4. Overexpression of Four Candidate Genes and Its Promising Diagnostic Significance

**Figure 4:**
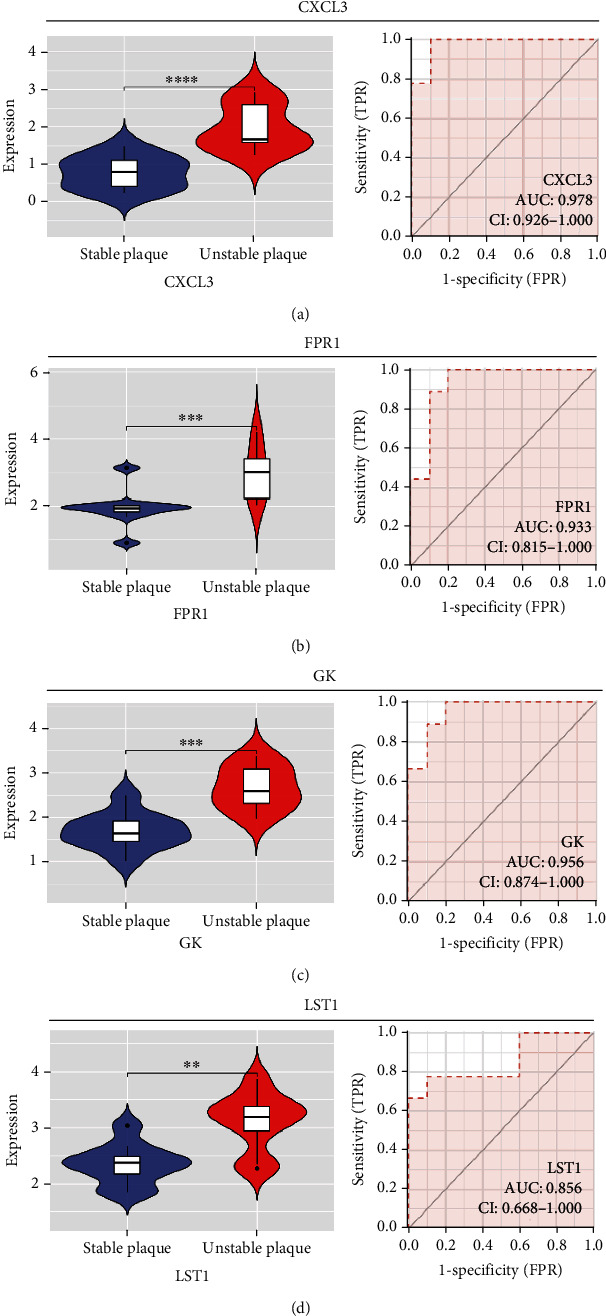
Expression level and diagnostic ability of the candidate genes in merged dataset. (a) CXCL3. (b) FPR1. (c) GK. (d) LST1 (^∗^*p* < 0.05, ^∗∗^*p* < 0.01, ^∗∗∗^*p* < 0.001, ^∗∗∗∗^*p* < 0.0001).

**Figure 5:**
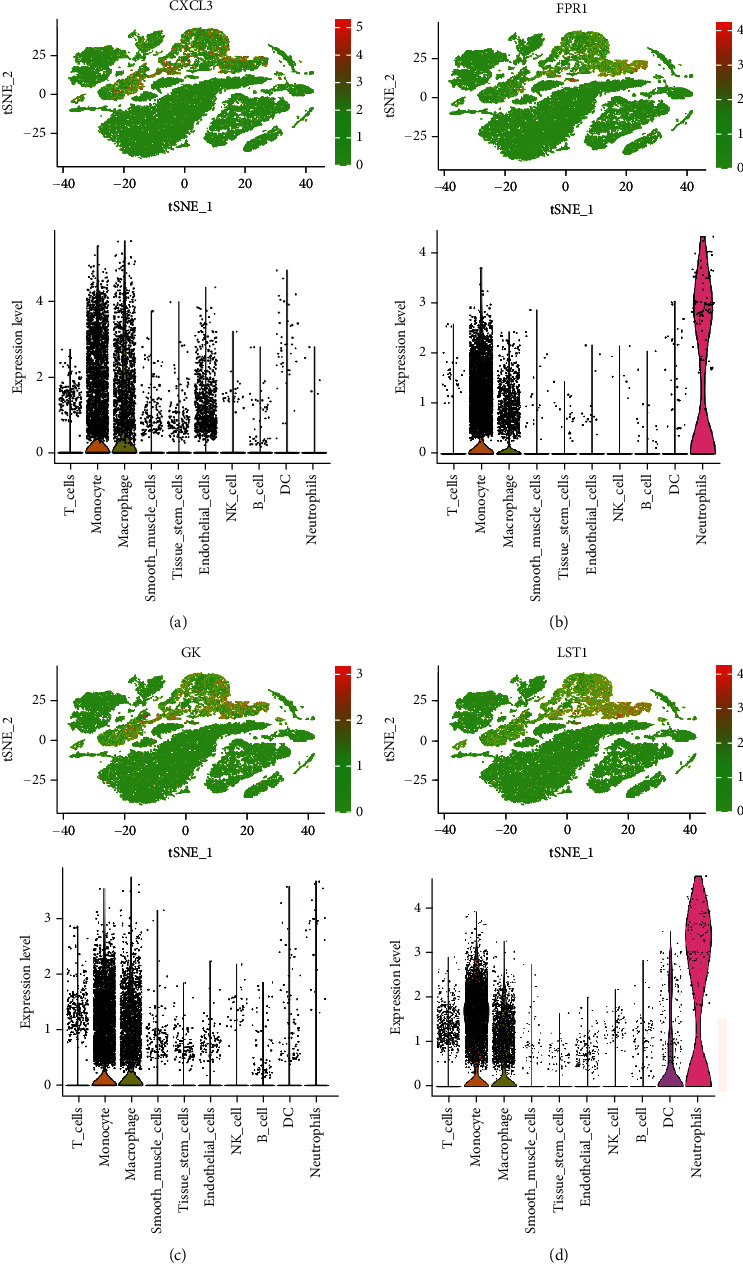
The expression level of the candidate genes at the single-cell level was shown in t-SNE and violin plots. The redder the color represented the higher expression level. (a) CXCL3. (b) FPR1. (c) GK. (d) LST1.

In our study, we determined the expression status of four candidate genes (CXCL3, FPR1, GK, and LST1) and corresponding diagnostic significance in unstable plaques. Our study showed that these candidate genes had moderate and above discriminative capability and could effectively discriminate between stable and unstable plaques. Besides, we detected the overexpression of the candidate genes in unstable atherosclerotic plaques in the merged dataset (Figures 4(a)–4(d)). Not only that, but we also detected their upregulated expression in monocytes (Figures 5(a)–5(d)). In conclusion, our study suggested that the expression of four candidate genes was significantly upregulated, which has a certain diagnostic value for unstable plaques.

### 3.5. Functional Enrichment Analysis of DEGs

**Figure 6:**
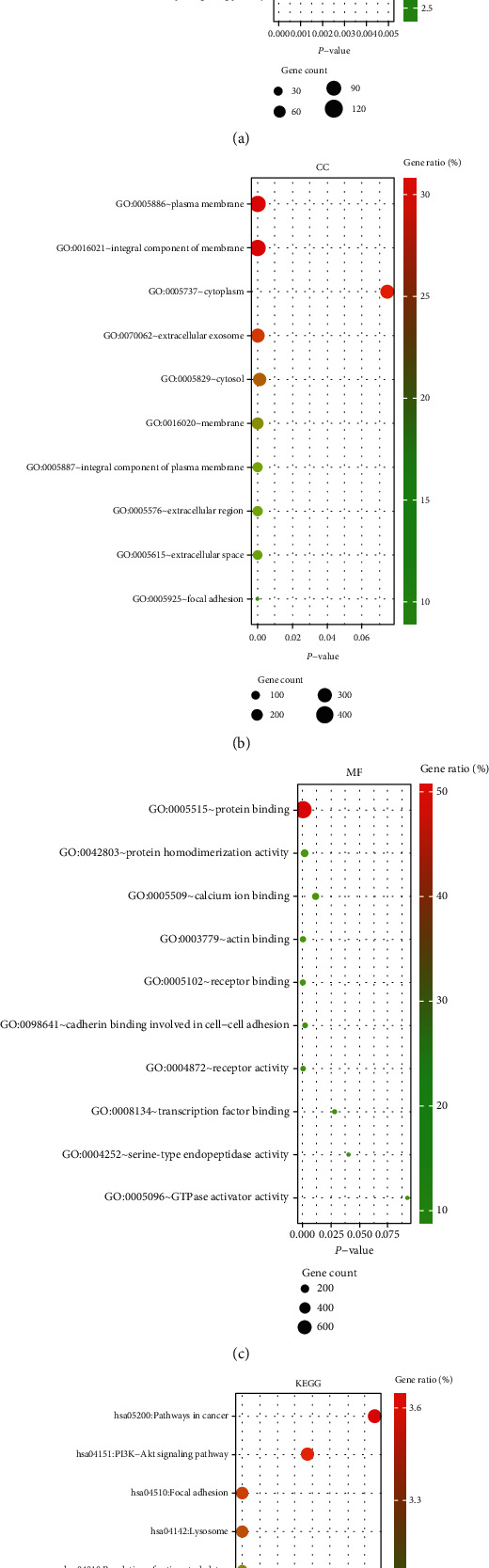
Functional enrichment analysis of 1221 differentially expressed genes (DEGs) by DAVID database: (a) biological process (BP); (b) cellular component (CC); (c) molecular function (MF); (d) KEGG pathway analysis.

We performed GO enrichment analysis and KEGG pathway analysis through the online DAVID database. GO enrichment analysis had three parts, including cellular components (CC), biological process (BP), and molecular function (MF). For BP, DEGs were mainly enriched in signal transduction, inflammatory response, immune response, etc. (Figure 6(a)). Regarding CC, DEGs were mainly enriched in the plasma membrane, integral component of membrane, etc. (Figure 6(b)). In terms of MF, DEGs were significantly enriched in protein binding, protein homodimerization activity, etc. (Figure 6(c)). In addition, KEGG pathway analysis showed that these genes were particularly related to the PI3K-Akt signaling pathway, pathways in cancer, and focal adhesion (Figure 6(e)).

### 3.6. Gene Set Enrichment Analysis (GSEA)

**Figure 7:**
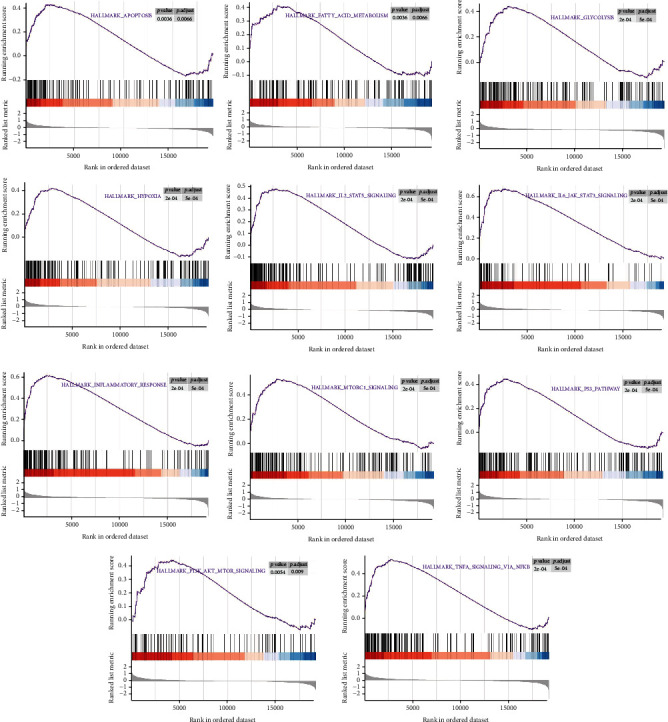
GSEA plot showed that multiple signaling pathways may be involved in the regulation of plaque instability, including “HALLMARK_INFLAMMATORY_RESPONSE” and “HALLMARK_PI3K_AKT_MTOR_SIGNALING.”

Herein, GSEA was carried out to explore underlying pathways involved in the development of unstable plaques, which may participate in the regulation of plaque instability. Our results suggest that multiple signaling pathways may be involved, including “HALLMARK_INFLAMMATORY_RESPONSE” and “HALLMARK_PI3K_AKT_MTOR_SIGNALING” (Figure 7).

### 3.7. Identification and Functional Analysis of Overlapping Coexpressed Genes (CEGs) of Candidate Genes

**Figure 8:**
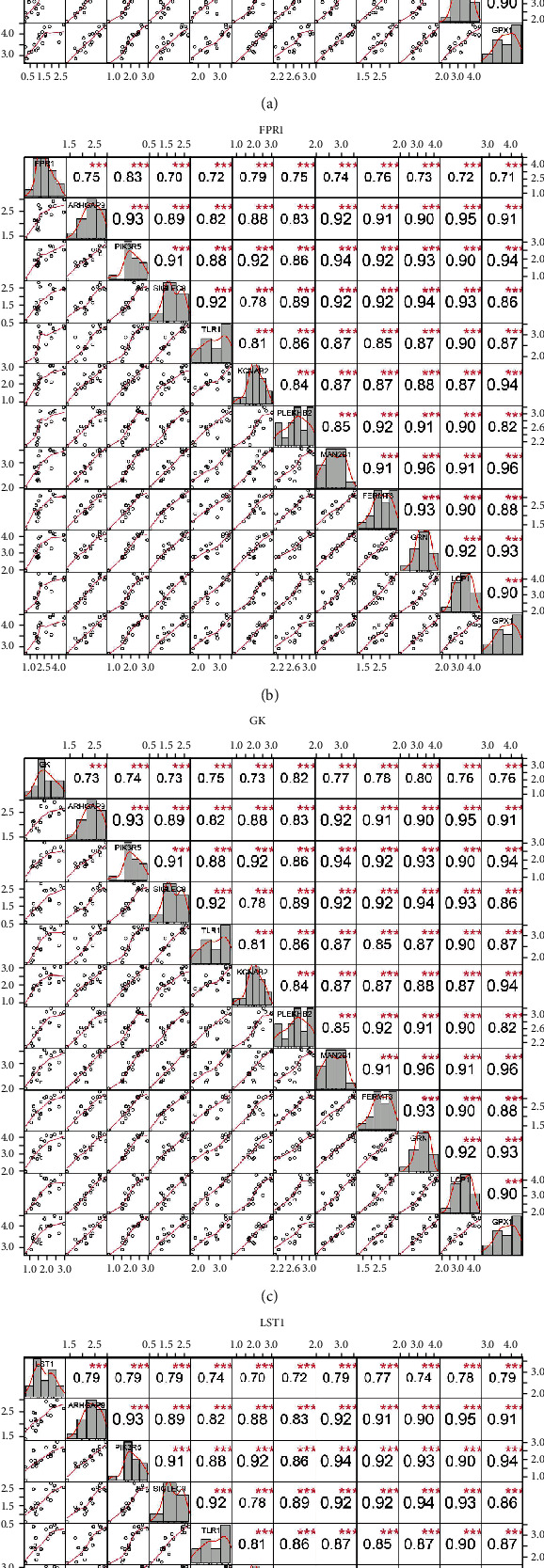
Correlations between the candidate genes and 11 overlapping coexpressed genes (CEGs): (a) CXCL3; (b) FPR1; (c) GK; (d) LST1.

**Figure 9:**
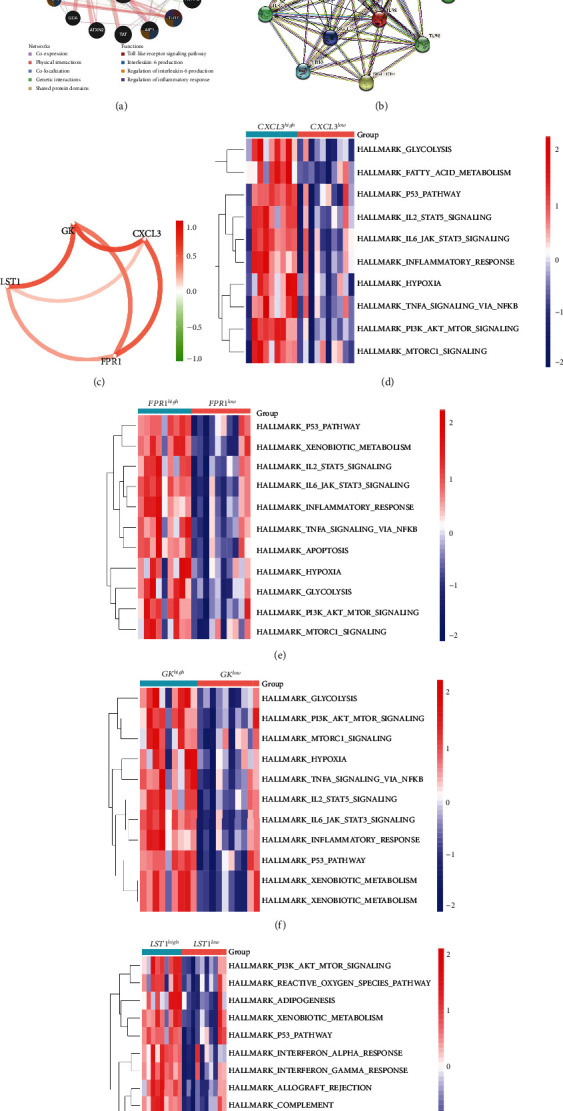
(a) Functional analysis of 11 coexpressed genes from the GeneMANIA database. (b) Protein interaction analysis network diagram of related genes on STRING database. (c) Correlation between the candidate genes. (d–g) Four heatmaps showed the results of gene set variation analysis (GSVA) of the candidate genes, arranged by CXCL3, FPR1, GK, and LST1.

We identified 11 overlapping CEGs of the candidate genes (CXCL3, FPR1, GK, and LST1) in the merged dataset.  Figure 8 shows the correlation between the candidate genes and 11 overlapping CEGs. Further, functional analysis from the GeneMANIA database suggested that these genes were closely related to regulating inflammatory responses (Figure 9(a)). From the protein interaction analysis of STRING database, we can see that there is a close relationship between related genes (Figure 9(b)).

### 3.8. Gene Set Variation Analysis (GSVA) and Identification of Correlations between Candidate Genes

Our study found that there was a significant positive correlation between these candidate genes (CXCL3, FPR1, GK, and LST1) (Figure 9(c)). Then, we conducted GSVA to explore signaling pathways associated with the candidate genes in unstable plaques. This study revealed that multiple pathways, including “HALLMARK_PI3K_AKT_MTOR_SIGNALING” and “HALLMARK_INFLAMMATORY_RESPONSE,” were significantly activated in the samples with high expression of the candidate genes (Figures 9(d)–9(g)). Notably, this result was consistent with that of the GSEA.

### 3.9. Evaluation of Protein Interaction and Correlation Analysis of PIK3R5 Gene

**Figure 10:**
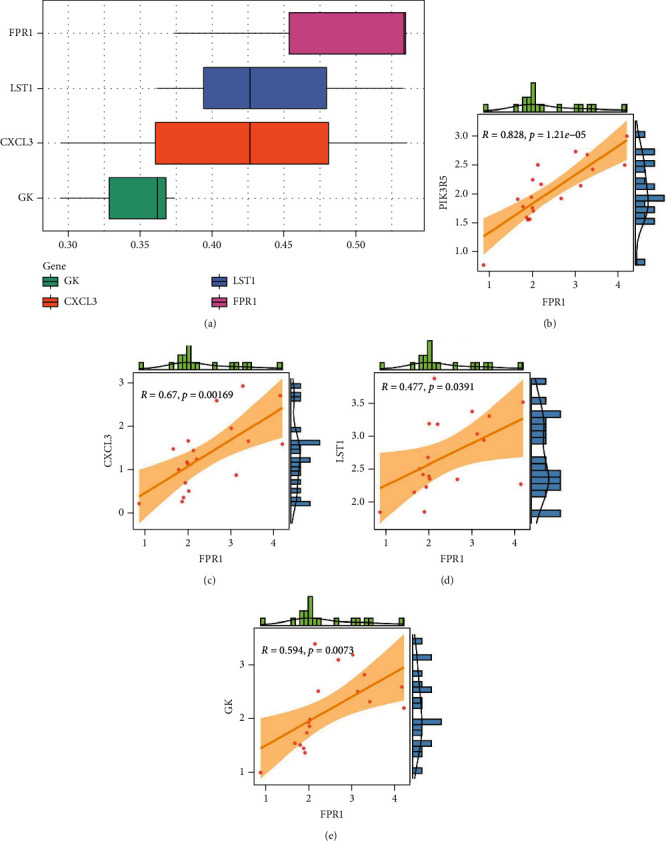
(a) Summary of protein functional similarity of overlapping genes. (b–e) Correlation analysis between FPR1 gene and PIK3R5 and other overlapping genes.

We analyzed each candidate gene at the level of biological process (BP) and cellular component (CC) and got the protein similarity score of the candidate genes. In the box diagram, the line in the box indicates the median value of functional similarity, and it can be seen that the score of FPR1 is the highest (Figure 10(a)). In the correlation analysis diagram, FPR1 is positively correlated with PIK3R5 and other overlapping genes significantly (Figures 10(b)–10(e)).

## 4. Discussion

Atherosclerosis (AS) is an occlusive artery disease that usually occurs in middle-aged and older adults, which seriously threatens patients' health and life quality [24, 25]. In particular, unstable atherosclerotic plaques rupture, leading to blood embolism, which can lead to a series of serious clinical consequences that can be fatal for patients [26, 27]. It is therefore critical to explore biomarkers associated with plaque instability and to take targeted interventions and preventive measures to reduce fatal clinical complications. In this study, we sought to combine single-cell sequencing data with transcriptome data to elucidate the clinical significance and potential mechanisms of monocyte-associated genes in atherosclerotic plaque instability.

As we all know, monocyte recruitment in the vascular wall plays an important role in the development of atherosclerosis [28, 29]; however, there are relatively few studies about the role of monocyte-associated genes on atherosclerosis. Our current study identified four (CXCL3, FPR1, GK, and LST1) significant monocyte-associated candidate genes related to atherosclerotic plaque instability using single-cell sequencing data analysis, WGCNA analysis, and differential expression analysis methods. We found that these candidate genes were significantly overexpressed in unstable plaques compared with stable plaques. Single-cell sequencing data analysis indicated that the expression of these genes was upregulated in monocytes. There are some other studies that support our findings. Gargalovic et al. found that CXCL3 might contribute to the inflammatory effects of oxidized1-palmitoyl-2-arachidonyl-sn-3-glycero-phosphorylcholine in the atherosclerotic lesion, which is crucial for the recruitment of monocytes [30]. Autoradiography and immunohistochemical analysis in one study has confirmed abundant FPR1 expression in atherosclerotic lesions [31]. Many studies have shown that GK is an essential enzyme in the formation of triacylgycerol [32, 33], which contributes to the development and progression of atherosclerosis [34, 35]. Furthermore, it was found that the candidate genes have the upper-middle ability to distinguish the stable and unstable plaques (AUC > 0.850 for all), which suggests that the four upregulated monocyte-associated candidate genes may be underlying biomarkers for early identification and intervention of unstable plaques.

Meanwhile, our study was also devoted to exploring the underlying mechanisms of the candidate genes in atherosclerotic plaque instability. Previous studies have reported that CXCL3, FPR1, and LST1 were closely associated with inflammation and oxidative stress, while GK played a central role in adipogenesis and gluconeogenesis [25–28]. CXCL3 is a well-known proinflammatory gene [36, 37]. FPR1 serves as a receptor that mediates oxidative stress signaling in the mammalian cell [38, 39, 40]. A study found that LST1 regulates inflammatory response in a model of inflammatory disease [41]. As for GK, it also shows a potential relationship with oxidative stress [42]. Previous studies have reported that these candidate genes were closely associated with inflammation and oxidative stress. It is consistent with our previous study that a high functional similarity was found among the candidate genes, which indicates that these candidate genes may act on several similar pathways. In our study, GO and KEGG analyses suggested that DEGs might be related to inflammation response and the PI3K-Akt signaling pathway. GSEA indicated that multiple pathways, including “HALLMARK_PI3K_AKT_MTOR_SIGNALING” and “HALLMARK_INFLAMMATORY_RESPONSE,” were excessively activated in unstable plaque. GSVA showed that these two pathways were also significantly activated in high expression samples of the candidate genes. Furthermore, coexpression analysis suggested that these candidate genes may be related to regulating inflammatory response. In conclusion, existing evidence showed that the candidate genes were likely to affect the atherosclerotic plaque instability through the inflammatory response and PI3K/Akt/mTOR signaling pathways, a well-known pathway relating to oxidative stress. Previous studies have shown that inflammation played an important role in the progression of atherosclerosis, which may be involved in driving plaque instability [43–46]. Another study showed that it could affect the initial recruitment of white blood cells to the final rupture of unstable atherosclerotic plaques [47]. In addition, numerous studies have reported its crucial role in the rupture of atherosclerotic plaque [48–51]. Similarly, our current study also indicated that the candidate genes were closely related to the excessive activation of inflammatory response in unstable plaques, which may be one of the potential mechanisms that aggravated the instability of atherosclerotic plaques. From the existing evidence, we speculate that the crosstalk between the candidate genes and PI3K/Akt/mTOR signaling pathway may activate inflammatory response leading to atherosclerotic plaque instability.

However, it was undeniable that our study conclusions may also have certain limitations due to the insufficient sample size, which required further in vivo and in vitro experimental verification.

## 5. Conclusions

Taken together, this study shows that the upregulated CXCL3, GK, FPR1, and LST1 may be effective biomarkers for early identification and intervention of unstable atherosclerotic plaques. Besides, we speculate that they may play a synergistic role with CEGs in regulating inflammation and PI3K/AKT/mTOR signaling pathways, thereby aggravating the instability of atherosclerotic plaques and promoting plaque rupture.

## Data Availability

All the data used to support the findings of the study can be found in the article.
